# An Efficient Distributed Algorithm for Constructing Spanning Trees in Wireless Sensor Networks

**DOI:** 10.3390/s150101518

**Published:** 2015-01-14

**Authors:** Rosana Lachowski, Marcelo E. Pellenz, Manoel C. Penna, Edgard Jamhour, Richard D. Souza

**Affiliations:** 1 PPGIa, Pontifical Catholic University of Parana – Parana, Curitiba 80215-901, Brazil; E-Mails: rosana.lachowski@pucpr.br (R.L.); penna@ppgia.pucpr.br (M.C.P.); jamhour@ppgia.pucpr.br (E.J.); 2 CPGEI, Federal University of Technology – Parana, Curitiba 80230-901, Brazil; E-Mail: richard@utfpr.edu.br

**Keywords:** wireless sensor networks, distributed spanning tree algorithms, routing, shortest path trees

## Abstract

Monitoring and data collection are the two main functions in wireless sensor networks (WSNs). Collected data are generally transmitted via multihop communication to a special node, called the sink. While in a typical WSN, nodes have a sink node as the final destination for the data traffic, in an *ad hoc* network, nodes need to communicate with each other. For this reason, routing protocols for *ad hoc* networks are inefficient for WSNs. Trees, on the other hand, are classic routing structures explicitly or implicitly used in WSNs. In this work, we implement and evaluate distributed algorithms for constructing routing trees in WSNs described in the literature. After identifying the drawbacks and advantages of these algorithms, we propose a new algorithm for constructing spanning trees in WSNs. The performance of the proposed algorithm and the quality of the constructed tree were evaluated in different network scenarios. The results showed that the proposed algorithm is a more efficient solution. Furthermore, the algorithm provides multiple routes to the sensor nodes to be used as mechanisms for fault tolerance and load balancing.

## 1. Introduction

Wireless sensor networks are composed of a set of tiny autonomous nodes deployed with the purpose of collecting information from a supervised environment [1,2]. Data collected by sensors are usually transmitted via multihop communication to the sink. It is assumed that the sink node has unlimited computational resources. This operation is named convergecast in the literature [3,4]. There are many examples of convergecast applications: smart environments, habitat monitoring, monitoring of areas that present risks to humans (volcanoes, seismic instability regions, regions susceptible to the occurrence of hurricanes), precision agriculture, traffic control, *etc.*

Convergecast uses a many-to-one communication model, while a typical *ad hoc* network uses a many-to-many communication model. Therefore, routing protocols for *ad hoc* networks are inefficient for WSNs. Trees, on the other hand, are traditional routing structures widely used in WSNs and present several advantages [5]. Sensor nodes need only to forward the collected data to their parent node in the routing structure and, thus, can easily select the appropriate transmission power to reduce power consumption. There is no need for a frequent route discovery mechanism, and sensor nodes do not have to maintain a table to store all routing paths. Moreover, trees are also the most appropriate structure for TDMA-based WSNs and for the use of a data aggregation strategy [3].

### 1.1. Motivation

Recently published works investigate the quality of the routing trees in WSNs in order to extend network lifetime. These improvements include balancing the tree to distribute the load among nodes [4] and using specific metrics (residual energy, link quality, proximity to the sink) to construct the routing paths [1]. Little attention has been paid to efficient tree construction. Traditionally, the efficiency of distributed algorithms is measured by runtime and the number of messages exchanged among the nodes [6]. Runtime is an important metric for WSNs, because some applications require a fast reconstruction of the routing structure if sensor nodes fail or exhaust their batteries. For instance, in safety and mission-critical applications where sensor nodes are deployed to detect oil/gas leaks or structural damage, delays can cause unexpected and catastrophic events [3]. Moreover, if the network requires frequent reconfiguration, the routing tree may not have enough time to be updated. The number of messages exchanged among nodes is another important metric, because most of the energy of sensor nodes is consumed by data communication [7–9]. As sensor nodes are usually battery-powered, energy is a very crucial resource.

Shortest path trees (SPTs) are the most common routing trees in the many-to-one networks and particularly in WSNs, because they represent an efficient routing structure in terms of time and energy [1,9]. There are many algorithms to construct SPTs, including the algorithms of Dijkstra [10] and Bellman–Ford [11]. However, these algorithms are centralized solutions that must be avoided, because of the unacceptable communication overhead involved in network topology discovery, since sensor nodes have scarce computational resources. Moreover, WSNs in general do not have centralized management and fixed infrastructure. In [12], Bertsekas and Gallager proposed a distributed asynchronous version of the Bellman–Ford algorithm for distributed systems. The distributed Bellman–Ford (DBF) presents fast convergence, the absence of any synchronization overhead and easy adaptation to topological network changes, but it is not efficient for networks composed of a large number of nodes or dense networks, because of the excessive number of messages required for the tree construction [13]. In [9], the authors propose an algorithm called shortest hop multipath (SHM) in order to overcome the communication overhead of the DBF and to construct multiple paths between nodes and the sink to be used as mechanisms for fault tolerance and load balancing. However, sensor nodes have to partially know the network topology, and the algorithm cannot construct the routing tree in the event of message losses or node failures. Message loss is a common event in wireless networks, and sensor nodes are prone to failures.

### 1.2. Contributions

In this paper, we propose an efficient distributed spanning tree algorithm suitable for WSNs. Our algorithm is based on Bellman–Ford distributed asynchronous version in order to inherit desirable qualities. The goal is to reduce the communication overhead of the DBF, making it a scalable solution without degrading the quality of the tree. Simulations show that the proposed algorithm can reduce both the number of messages required for the tree construction and runtime by approximately 87.5% compared to the DBF. In addition, the proposed solution constructs multiple paths as the SHM algorithm, being tolerant to message losses and node failures.

The remainder of the paper is organized as follows. In Section 2, we explain why distributed algorithms for constructing spanning trees proposed in the literature are unsuitable for WSNs. Section 3 presents the proposed algorithm. Simulation results are given in Section 4. Finally, Section 5 concludes the paper and outlines future research directions.

## 2. Related Work

The routing algorithms for WSNs can be classified as float or data-centric, hierarchical and geographic or location-based [14,15]. Data-centric protocols are query-based. The sink node sends queries to the network, and sensor nodes send data in response to these queries [15]. All sensors play the same role, and their duty is to send data packets to the sink. Geographic protocols utilize the geographic location of nodes and their position in the network to route data packets to the sink [16]. Position information is generally achieved by GPS (Global Positioning System) or by exchanging information among nodes.

Clustering protocols assign different tasks to the nodes. Clustering zone and parent-child relationships are commonly used [16]. In the clustering approach, the network is divided into groups, called clusters. Each cluster has a leader node, usually called the cluster head, which is responsible for collecting data from cluster members and routing data packets towards the sink. In the parent-child approach, the parent node is responsible for collecting data from its children nodes and forwarding the packets to its own parent. Packets are relayed from parent to parent, until they reach the sink.

In [15], routing protocols designed for WSNs are categorized according to their routing goal: energy conservation, fast delivery and fault tolerance. Energy-efficient approaches utilize two methods: energy balancing and energy consumption reduction. Delay-aware approaches are classified into two types: hard delay aware and soft delay aware. The main goal of hard delay-aware approaches is to deliver real-time data on-time, while the main goal of software delay-aware approaches is just to select the shortest path. Fault-tolerant approaches use two strategies against faults: using a method or different levels of redundancy to prevent failures or using a method to detect and to repair the failures.

The authors in [14] claim that hierarchical routing protocols are the main method to solve the energy problem in large-scale WSNs and categorize clustering protocols designed especially for large-scale WSNs according to their goals: control overhead reduction, energy consumption mitigation and energy balance. In addition, various clustering algorithms proposed in the literature were compared using the following metrics: message complexity, memory requirement, localization, data aggregation, clustering manner, intra-cluster topology, cluster-head election and multipath routing. Message complexity, which represents the number of messages exchanged among nodes, directly affects the scalability of the routing protocol. Therefore, routing protocols designed for large-scale WSNs must consider reducing the message complexity. In order to enhance the scalability, routing protocols must also efficiently utilize the limited memory capacity of sensor nodes. The utilization of GPS must be avoided, since extra hardware increases economic costs. Data aggregation strategies enhance energy efficiency and scalability, since the number of messages in transit on the network is reduced.

Another way to enhance energy efficiency is to construct the network clusters on demand and to use the topology of a spanning tree intra-cluster. The authors in [14] provide some examples of clustering algorithms that use spanning trees to enhance scalability and to extend network lifetime. More energy-efficient LEACHfor large-scale WSNs (MELEACH-L) [17] connects some nodes of the network by a spanning tree in order to construct a virtual backbone. The base station-controlled dynamic clustering protocol (BCDCP) [18] uses a similar strategy, in which the clusterheads are connected by a tree and the sink functions as the manager of the whole network. The dynamic minimal spanning tree routing protocol (DMSTRP) for large wireless sensor networks [19] improves BCDCP and constructs a spanning tree intra-cluster and inter-cluster. The protocol uses a data fusion strategy along the tree route and minimizes collisions by arranging the transmission sequence for the nodes within a cluster. The use of spanning trees for routing and clustering in WSNs is an important issue, and it is the focus of our study.

The most common methods of constructing spanning trees are: depth first search (DFS) and breadth first search (BFS) [20]. The strategy followed by DFS is to visit the vertices reachable from a previous vertex, before visiting their adjacent vertices. The DFS method constructs trees with a few branches and leaves. The structure looks more like a path. Because of the tree depth, sensor nodes consume a lot of time and energy to send data to the sink. A large amount of time is also required to construct the DFS tree, since DFS is inherently sequential [21]. The strategy followed by BFS is to visit all adjacent vertices of a previous vertex before visiting other vertices. The BFS method constructs trees with many branches and consequently a small depth. BFS is the basis for algorithms that construct SPTs.

In the context of WSNs, an SPT is a spanning tree rooted at the sink, such that the cost from any node to the sink is minimal. The cost *cost*(*p*) of a path *p* = < *i, j, sink* > is the sum of the costs of its constituent links:

(1) $$\text{cost}\left(p\right)={\text{cost}}_{i,j}+{\text{cost}}_{j,\text{sin}k.}$$

Several different metrics have been proposed in the literature to compute the cost of the links [1]. Basic approaches use the number of hops and the distance to the sink. Distributed Bellman–Ford is used in [1,22,23] for constructing SPTs in WSNs. According to [12], the SPT is constructed by the iteration:

(2) $$W_{i}^{t+1}=\underset{j\in N_{i}}{\min}{\text{cost}}_{i,j}+W_{j}^{t},i\neq 1,$$

where *N_i_* represents the set of neighboring nodes of *i*, $W_{i}^{t}$ is the weight of node *i* in step *t, i.e.*, the last estimated cost of the path from *i* to the root node computed at node *i*, and *cost_i,j_* is the cost assigned to the link between nodes *i* and *j*. The initial conditions are $W_{1}^{0}=0$ and $W_{i}^{0}=\infty$ for *i* ≠ 1. The pseudocode of DBF is presented in Algorithms 1 and 2. The sink initiates the tree construction by sending a broadcast message with its cost (Algorithm 1, Step 2). When a node *i* receives the message containing the weight of the sending node *j*, it computes the path cost to the sink if it selects node *j* as the parent node. For doing so, node *i* adds the link cost to node *j* to the received weight. If the cost of the current route from node *i* to the sink exceeds the cost of the route that is being offered (Algorithm 2, Step 3), node *i* selects *j* as the temporary parent (Algorithm 2, Step 4), updates its weight (Algorithm 2, Step 5) and broadcasts a message with its new weight (Algorithm 2, Step 6). The algorithm ceases when there are no more messages in transit on the network. Bertsekas and Gallager [12] proved that the SPT is constructed even if some nodes are slower than others to propagate or calculate their weights.


**Algorithm 1:** Distributed Bellman–Ford (DBF), initiator node.
 1:*parent_sink_* = *sink*; *W_sink_* = 0 2:broadcast < *sink*,*W_sink_* >



**Algorithm 2:** DBF, non initiator node.
 1:*parent_i_* = *null*; *W_i_* = ∞ 2:**while** node *i* receives < *j,W_j_* > message from node *j* 3:**if**
*W_i_* > *W_j_* + *cost_i,j_*
**then** 4: *parent_i_* = *j* 5: *W_i_* = *W_j_* + *cost_i,j_* 6: broadcast < *i,W_i_* > 7:**end if** 8:**end while**


In [9], the authors argue that the DBF is unsuitable for WSNs because of its high energy cost and also because the sink cannot know whether the algorithm has terminated. To provide a suitable solution to construct SPTs in WSNs, the authors of [9] propose the SHM algorithm, which requires five types of messages (probe, acknowledgment (ack), pulse, pulseAck and pulseNack) and uses the hop count to the sink as the cost of the links. The algorithm is synchronous, and the sink is responsible for initializing and finalizing the spanning tree construction, which is performed in layers. To initiate the construction process, the sink broadcasts a message (probe) offering paternity and then waits for an acknowledgment message (ack) from each of its possible children. After receiving all expected messages, the sink broadcasts a message (pulse) in order to inform which layer must offer paternity. Nodes that offer paternity also wait for acknowledgments from their possible children to then unicast a message (pulseAck) to their parents. Sensor nodes that have no possible children do not offer paternity, but unicast a message (pulseNack) to inform their parents about their condition. If a node receives only pulseNack messages from its children as a reply for a pulse message, it unicasts a pulseNack message to its parent. Otherwise, the node unicasts a pulseAck message to its parent. These messages (pulseAck and pulseNack) are relayed from parent to parent, until they reach the sink. If the sink receives only pulseNack messages from its children as a reply for a pulse message, it knows that the algorithm execution finished and does not broadcast another pulse message. While the algorithm is executed, sensor nodes construct a set of alternative parents to be used for fault tolerance or load balancing.

In [24], the authors propose a distributed algorithm for depth first search. The algorithm is sequential and uses a backtrack strategy. Two types of messages are required: forward and return. The only difference in message treatment is that the forward message leads to the initialization of local variables. The root initiates the construction process by sending a forward message to itself. When *i* receives a forward or return message and it has unvisited neighbors, node *i* selects one of the unvisited neighbors *u* and adds to the set of child nodes. Then, *i* unicasts a forward message to *u*. If node *i* does not have unvisited neighbors, it unicasts a return message to *returnnode*, a local variable that represent an ancestor node that has unvisited neighbors. If *returnnode* is not a neighbor node, *i* unicasts a return message to its parent node. When the root receives a return message and it does not have unvisited neighbors, the algorithm execution terminates. Unlike the DBF and SHM algorithms, nodes do not select the parent node according to a specific metric. Therefore, the DFS algorithm demands a lower number of messages, but does not construct an SPT.

### 2.1. Efficiency Evaluation

**Figure 1.**
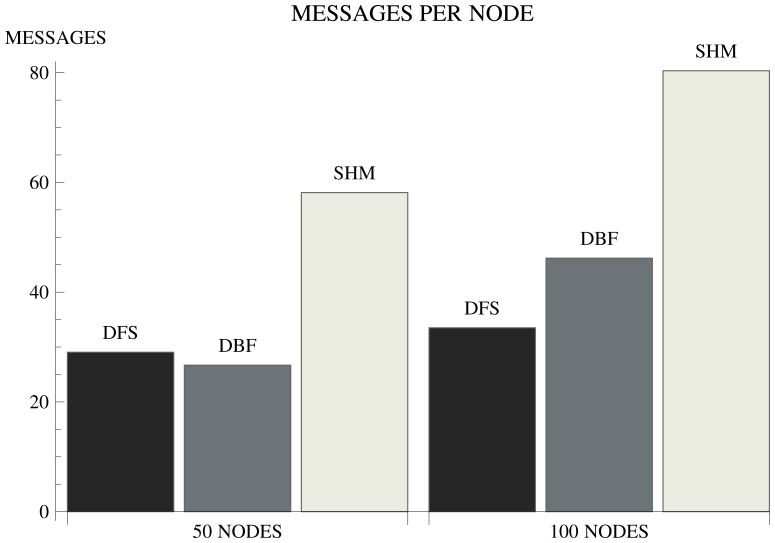
Average number of messages per node.

**Figure 2.**
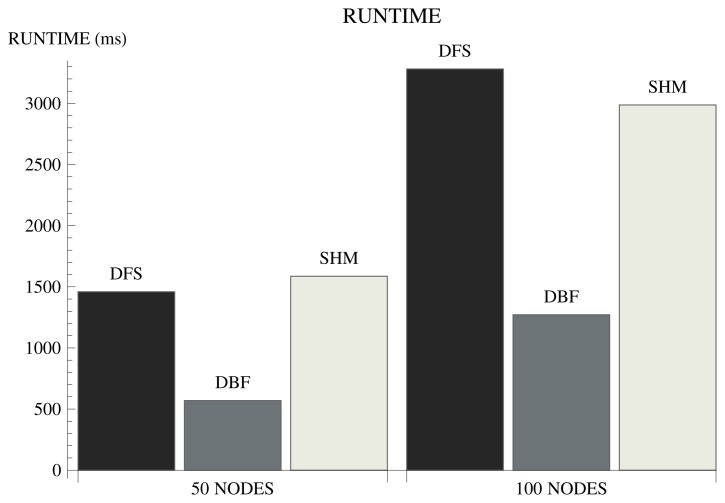
Runtime.

In order to evaluate the efficiency of some well-known distributed spanning tree algorithms, we implemented the DFS algorithm proposed in [24] and the DBF [12] and SHM [9] algorithms, which are based on the BFS method. The algorithms were simulated assuming an IEEE802.15.4-based [25] WSN. The simulator considers the typical radio parameters to determine the transmission and interference ranges and implements the CSMA/CA MAC strategy. Further details about the simulation framework are presented in Section 4.1. Two scenarios are considered involving network topologies formed by 50 and 100 nodes. The metrics are the number of messages required for the tree construction and the runtime of the algorithms, whose results are shown in Figures 1 and 2.

Among the evaluated algorithms, DBF presents the shortest runtime. However, the performance with respect to the number of messages considerably degrades with the increasing number of nodes. In a network formed by 50 nodes, DBF requires approximately 25 messages per node for the tree construction. In a network formed by 100 nodes, the number of messages increases by approximately 80%. WSNs are generally formed by a large number of nodes, which makes DBF an unsuitable solution. The advantage of DBF is simplicity: it demands few computational resources and does not require network topology knowledge.

In order for the sink node to know whether the algorithm terminated, SHM requires a complex synchronization mechanism. For this reason, the algorithm presents low efficiency with respect to the number of messages and runtime. Compared to DBF, the runtime of SHM increases approximately 130%. Furthermore, sensors have to know which nodes are one-hop away from them. The algorithm is inflexible with respect to the metric used to compute the cost of the links. However, the main drawback of SHM is the fact that it is not prepared for message losses or node failures during the SPT construction. A parent node can remain indefinitely waiting to receive a message from one of its children to continue the execution of the algorithm. The advantage of SHM is a resilient tree, since sensor nodes have alternative parents. If the parent node fails or dies, children nodes have alternative routes to send the collected data to the sink. The alternative routes can also be used as mechanisms for load balancing.

The DFS algorithm is a scalable solution with respect to the number of messages. Comparing the scenarios with 50 and 100 nodes, the number of messages increases by approximately 13%. However, the performance with respect to the runtime considerably degrades when the number of nodes increases. Comparing the scenarios with 50 and 100 nodes, the runtime increases by approximately 125%. The reason for the poor performance with respect to runtime is the fact that DFS is a sequential algorithm [21]. For the same reason, DFS is not prepared for message losses or node failures and requires sensors to know which nodes are one hop away from them. Table 1 summarizes the main features of the evaluated algorithms.

## 3. Proposed Algorithm

The design of the proposed algorithm was guided by the following goals:

- Low communication overhead: excessive message exchange reduces network lifetime;
- Simplicity: the algorithm must demand few computational resources and must not require network topology knowledge;
- Scalability: the solution must be efficient for networks composed of large number of nodes;
- Distributed approach: centralized solutions are unsuitable for WSNs;
- Suitable for wireless networks: the algorithm must construct the routing structure even in the event of message losses or node failures;
- Resilience: the solution must provide multiple paths to the sink;
- Quality of the tree: the quality of the constructed tree must be at least sub-optimal.

Our algorithm is called efficient Bellman–Ford (EBF), and it is based on the Bellman–Ford distributed asynchronous version in order to inherit certain characteristics: simplicity and tolerance to message losses or node failures during the execution of the algorithm. DBF is tolerant to message losses and node failures, because messages are always broadcast and the algorithm does not require any complex synchronization. Therefore, nodes never remain indefinitely waiting for a message to continue the execution of the algorithm, and hence, a reliable message delivery strategy is unnecessary Moreover, only a single message is enough for a node to select its parent in the routing structure and to keep the network connected. Logically, the greater the number of lost messages, the lower the quality of the constructed tree. As with DBF, the proposed EBF demands few computational resources, and sensors do not need to know their neighbors. The quality of the trees constructed by both algorithms is evaluated utilizing as metrics the average distance and hop count with respect to the sink. These metrics are directly affected by the number of lost messages during the algorithm execution. Thus, the larger the number of lost messages, the worse the quality of the constructed tree.

The excessive message exchange is the main drawback of the DBF algorithm. The more connected the network and the larger the number of sensors, the worse is the performance of DBF. The reason for the low performance in this aspect is the use of the flooding approach. Nodes accept and retransmit to all of their neighbors any paternity offer more advantageous than the offer accepted earlier in terms of the metric to construct the tree.

The main difference of EBF with respect to DBF is the inclusion of a strategy to reduce both the number of messages required to construct the tree and the runtime, making it practical for WSNs. Nodes accept and retransmit an offer only if its advantage over the offer previously accepted is greater than or equal to a factor *α*, 0 < *α <* 1. The value of *α* determines how much a new offer must be more advantageous than the current accepted offer in order to replace it. The value of *α* must be chosen so that the quality of the tree is not considerably affected. When *α* = 0, EBF is equivalent to DBF. Even though we chose *α* = 0.1 for the comparisons between DBF and EBF, computer simulations show that values between 0.06 and 0.1 are appropriate. The choice of a particular *α* depends on the application and on the goal that one intends to reach. The larger the value of *α*, the larger the reduction in the number of messages and in the execution time; however, the quality of the constructed tree decreases with *α*.

With respect to the multiple paths, the strategy is to make sensor nodes store all received paternity offers in order to construct a set of alternative parents. This strategy is inspired by the SHM algorithm. If a parent candidate sends multiple offers, only the last one is stored. Similarly to the DBF and assuming that Node 1 is the sink node, the construction of the SPT is given by iteration:

(3) $$W_{i}^{t+1}=\underset{j\in N_{i}}{\min}{\text{cost}}_{i,j}+W_{j}^{t},i\neq 1,$$

starting from the initial conditions $W_{1}^{0}=0$ and $W_{i}^{0}=\infty$ for *i* ≠ 1 and subject to the constraint:

(4) $$ad\upsilon_{i,j}\geq \alpha ,$$

where parameter *adv_i,j_* is the advantage of the route offered by *j* with respect to the cost of the current route of node *i* and can be calculated by:

(5) $$ad\upsilon_{i,j}=\left(W_{i}^{t}-\left({\text{cost}}_{i,j}+W_{j}^{t}\right)\right)/W_{i}^{t}.$$

Algorithms 3 and 4 present the EBF pseudocode. The sink initiates the tree construction by broadcasting a message with its cost (Algorithm 3, Step 2). When a node *i* receives the message containing the weight of the sending node *j* and it does not have a parent node, node *i* performs the following steps, represented in Algorithm 4:

(a) Selects *j* as temporary parent (Step 4);
(b) Updates its weight (Step 5);
(c) Broadcasts a message with its new weight (Step 6).

**Algorithm 3:** Efficient Bellman–Ford (EBF), initiator node.
 1:*parent _sink_* = *sink; W_sink_* = 0 2:broadcast < *sink, W_sink_* >



**Algorithm 4:** EBF, non initiator node.
 1:*parent_i_* = *null; W_i_* = ∞; AP*_i_* = ∅ 2:**while** node *i* receives < *j, W_j_* > message from node *j* 3: **if**
*parent_i_* = *null*
**then** 4: *parent_i_* = *j* 5: *W_i_* = *W_j_* + *cost_i,j_* 6: broadcast < *i, W_i_* > 7:**else** 8: **if** ∃(*j*, *x*) | *x* ∈ ℜ Λ (*j, x*) ∈ AP*_i_*) **then** 9:  AP*_i_* = AP*_i_\*{(*j, x*)} 10:**end if** 11:**if** (*W_i_* − (*W_j_* + *cost_i,j_*))*/W_j_* ≥ α **then** 12:  **if**
*parent_i_* ≠ *j*
**then** 13:   AP*_i_* = AP*_i_* U {(*parent_i_, W_i_*)} 14:   *parent_i_* = *j* 15:  **end if** 16:  *W_i_* = *W_j_* + *cost_i,j_* 17:  broadcast < *i, W_i_* > 18: **end if** 19: **if**
*parent_i_* ≠ *j*
**then** 20:  AP*_i_* = AP*_i_* U {(*j, W_j_* + *cost_i,j_*)} 21: **end if** 22:**end if** 23:**end while**


Otherwise, if node *i* already has a parent node, it adds the link cost to the node *j* to the received weight and computes the advantage of the new route with respect to the current route. If the advantage of the new route is at least equal to *α* (Algorithm 4, Step 11), node *i* performs the following steps, represented in Algorithm 4:

(a) Checks if *j* is its parent. If *j* is not its parent, then node *i* adds the parent node to the set of alternative parents (Steps 12 and 13). The set of alternative parents of node *i* is denoted by *AP_i_*.
(b) Selects *j* as the temporary parent (Step 14). This check is required to ensure that the current parent node is not added to the set of alternative parents;
(c) Updates its weight (Step 16);
(d) Broadcasts a message with its new weight (Step 17).

However, if the new route is not considered sufficiently advantageous, node *i* simply adds *j* together with its weight plus the cost of the link (*i, j*) to the set of alternative parents (Steps 19 and 20). Inevitably *j* becomes the parent node or becomes part of the set of alternative parents. For this reason, the receiving node removes *j* from the set of alternative parents, if *j* is already part of the set, as soon as it receives the setup message (Steps 8 and 9).

## 4. Simulation Results

Simulations are divided into two steps. The first step aims to empirically determine the optimal *α* that minimizes the number of messages and runtime for the construction of the routing tree, while maintaining the quality of the structure. The second step aims to compare the efficiency of the EBF and DBF algorithms and the quality of the constructed trees. The efficiency of the algorithms SHM and DFS are not compared, because they are infeasible for real-world scenarios, since they cannot construct the routing tree in the case of message losses or node failures. In addition, DFS does not construct an SPT

### 4.1. Network Modeling

The following assumptions are made about the network:

1. Each node has a distinct identification;
2. Links between nodes are symmetric; if there is a link from *i* to *j*, there is a reverse link from *j* to *i*;
3. Nodes do not know their geographical position;
4. The sink is located at (0,0), the origin;
5. Sensor nodes are equal in terms of processing power, radio, battery and memory, except the sink node;
6. The CSMA/CA (carrier sense multiple access/collision avoidance) protocol is used for medium access.

We model the WSN as a connected graph *G* = (*V, E*). A graph *G* = (*V, E*) consists of a finite nonempty set *V* of vertices and a finite set *E* ⊆ *V* × *V* of arcs [20]. Vertices are simple objects that can have names and attributes, and edges are connections between two vertices. Sets *V* and *E* represent, respectively, the node set and the link set. There is an edge (*i,j*) if the maximum transmission radius *r* of both *i* and *j* is at least their Euclidean distance ((*i,j*) ⇔ |*dist_i,j_|* ≤ *r*). The average degree $k=\frac{2_{*}\left|E\right|}{\left|V\right|}$ is the relation between the number of edges and the number of nodes. We consider grid network topologies with random disturbance in the position of nodes. To perform the simulations, we used the software, Mathematica [26], along with the SensorSim library [27]. The SensorSim library uses the transmission parameters of the IEEE 802.15.4 [25].

### 4.2. Simulation Setup

During the first step of the simulations, the parameters assume the following values: *α* ∈ {0.01, 0.02, 0.04, 0.06,0.08, 0.1, 0.2, 0, 4}, number of nodes *N* = 100 and network average degree *k* ∈ {4,6,8,10}. During the second step of the simulations, the parameters assume the values: *α* = 0.1, *N* ∈ {50,100,150, 200, 250, 300} and *k* = 8. For each number of nodes in the network, we considered a set of 10 randomly-generated topologies. The following metrics are used: number of messages required for the tree construction, runtime of the algorithm, average distance to the sink and average number of hops to the sink. The first two metrics (number of messages and runtime) are intended to evaluate the efficiency of the algorithms. Messages retransmitted due to collisions are not accounted for. The number of retransmissions is unlimited in order to create an ideal scenario to simplify the evaluation of the algorithms. The last two metrics (distance and number of hops) aim to evaluate the quality of the tree. The shorter the distance and the lower the number of hops from sensors to the sink, the higher the quality of the tree, because distance and number of hops impact the energy consumption and end-to-end delay during the operation of the network. Table 2 summarizes the simulation parameters. The transmission and interference range are based on typical IEEE 802.15.4 parameters along with the log-distance path loss model. We choose the distance between nodes to compute the cost of the links, because it is one of the methods most commonly used to relay messages in a wide variety of networks [9]. However, EBF and DBF algorithms can use any metric described in the literature. To estimate the distance to the sending node, the receiving node can use the RSSI (received signal strength indicator) [28].

### 4.3. First Step: Empirical Determination of Alpha

The first step of the simulations aims to define the value of *α* to be used by EBF for comparisons with DBF in the second step of the simulations.

**Figure 3.**
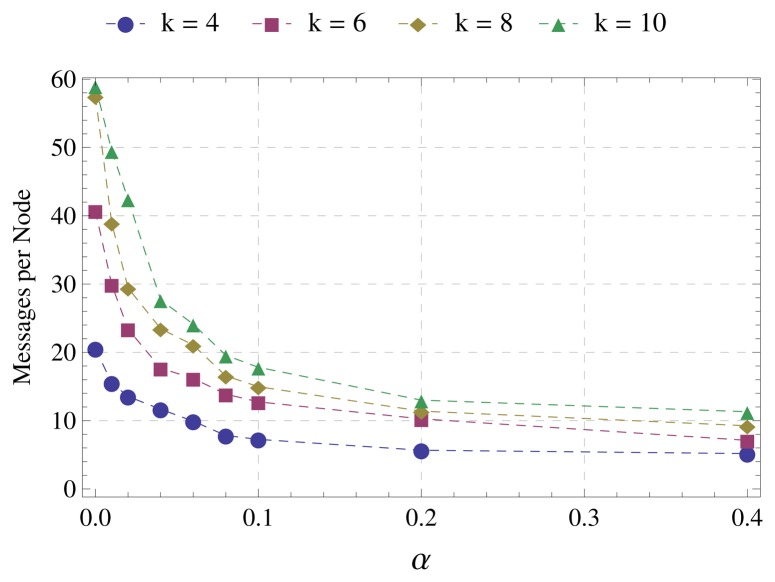
Average messages per node.

Figure 3 displays the average number of messages (sent and received) per node during the execution of EBF. The reduction in the number of messages is quite significant up to *α* = 0.1. From this point, the average number of messages tends to stabilize. It is also observed that the higher the network average degree *k*, the greater the reduction in the number of messages. This is because the performance of DBF worsens as the network becomes denser.

**Figure 4.**
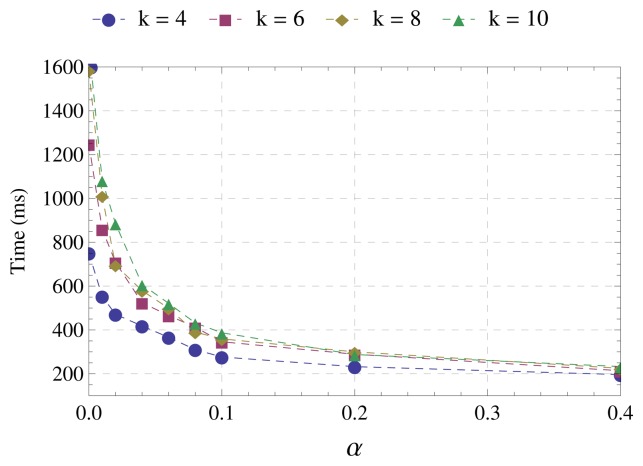
Runtime.

Figure 4 displays the time spent by the algorithm for the tree construction. Again, there is a significant reduction up to *α* = 0.1. Although this reduction is greater as the network average degree increases, from *α* = 0.1, the runtime is almost the same for all network degrees evaluated.

**Figure 5.**
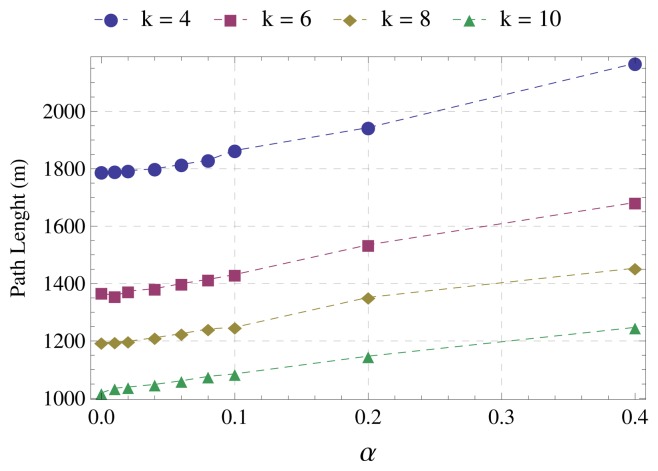
Average distance to the sink.

Figure 5 displays the average distance (in meters) from nodes to the sink. Up to *α* = 0.1, we can observe that the distance increases slightly. However, from *α* = 0.1 the increase in path length becomes more significant. We can also observe that this increase is more pronounced the lower the network degree. This is because the lower the network degree, the lower the number of possible paths. For the same reason, the distance to the sink increases as the network degree decreases. The graph shows that the increase in path length as a function of *α* is approximately linear, which suggests that we should use the lowest possible value of *α*.

**Figure 6.**
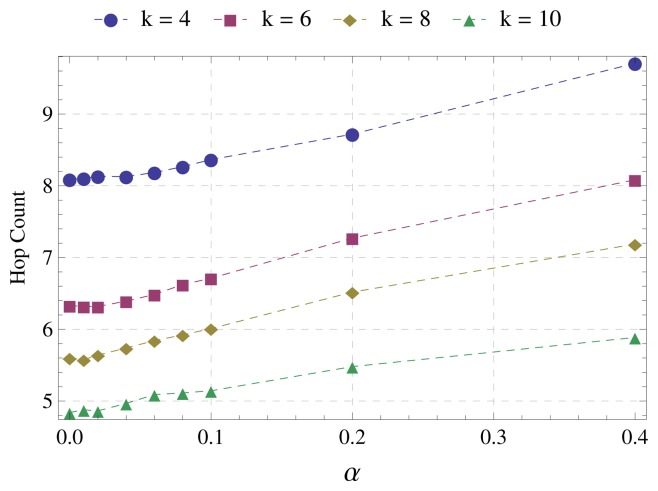
Average hops to the sink.

Figure 6 shows that *α* causes little impact on the average number of hops from sensor nodes to the sink. Up to *α* = 0.1, the difference does not exceed one hop and reaches a maximum of two hops when *α* = 0.4. We also identified that the parameter *α* does not influence the number of alternative parents, which is only affected by the network average degree. This is due to the operation of the algorithm. Nodes inevitably receive a paternity offer from all of their neighbors. Therefore, the denser the network, the greater the number of alternative parents. Parameter *α* influences only the number of subsequent messages and, thus, the distance of alternative parents to the sink.

Considering the above results, we set *α* = 0.1 for the comparisons between EBF and DBF. This choice is justified by the following observations:

- The most significant reduction in the number of messages occurs up to *α* = 0.1 and from this point tends to stabilize;
- The most expressive reduction in the runtime also occurs up to *α* = 0.1;
- The increase in the distance and number of hops from sensors to the sink is accentuated from α = 0.1.

### 4.4. Second Step: Algorithm Evaluation

The second step of the simulations aims to compare the efficiency of EBF and DBF and the quality of the constructed trees.

**Figure 7.**
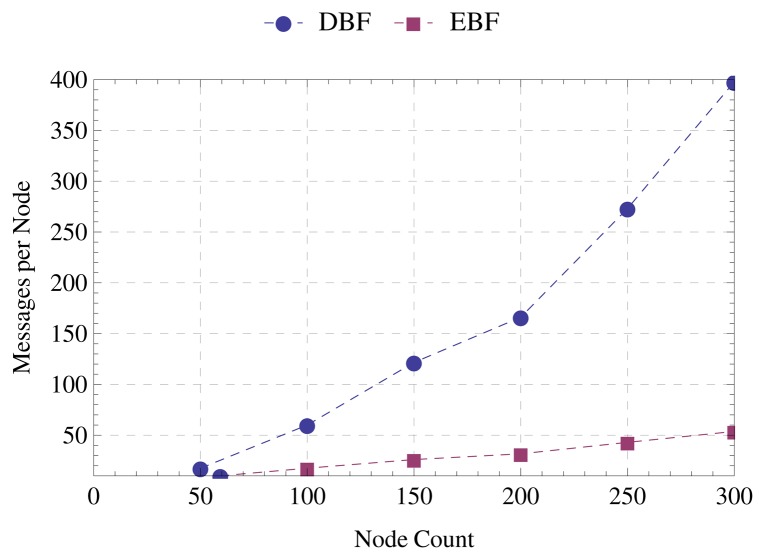
Average messages per node.

Figure 7 displays the average number of messages required by EBF and DBF for the tree construction. We can observe that the larger the number of nodes, the more significant is the difference between the number of messages required by the two algorithms. In a scenario with 50 nodes, /linebreak EBF requires 10 messages per node on average, while DBF requires approximately 20 messages per node; therefore a reduction of 50% using the proposed strategy. In a scenario with 300 nodes, DBF requires 400 messages per node on average, while EBF requires 50; thus a reduction of 87.5%. This means that as the number of nodes increases, the proportion of paternity offers less advantageous than *α* also increases. Comparing the scenarios with 50 and 300 nodes, the number of messages increases five-times for EBF and 20-times for DBF. Besides reducing communication overhead, EBF is also a more scalable solution.

**Figure 8.**
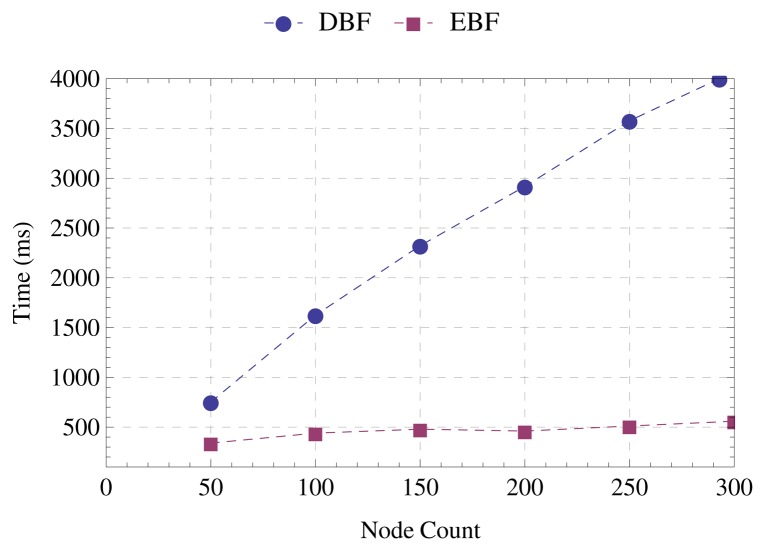
Runtime.

Figure 8 shows that the runtime of EBF is significantly lower than the runtime of DBF. In a scenario with 300 nodes, the percentage reduction in runtime and the number of messages is the same. Comparing the scenarios with 50 and 300 nodes, the runtime of EBF increases by about 37.5%, while the runtime of DBF increases by about 434%.

**Figure 9.**
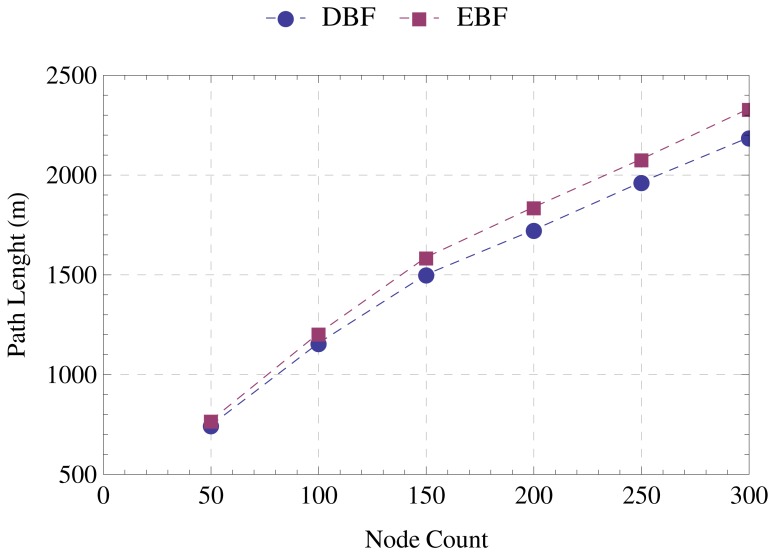
Average distance to the sink.

**Figure 10.**
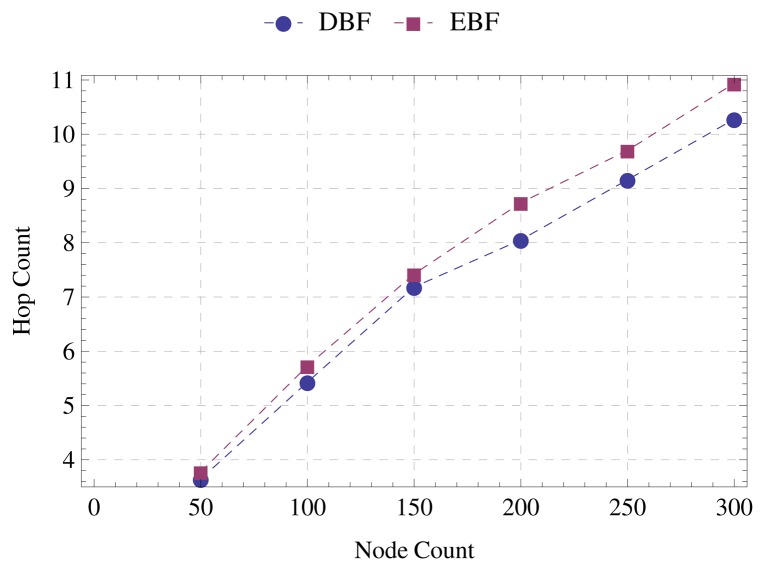
Average hops to the sink.

Figure 9 shows that the reductions in the number of messages and runtime have little impact on the quality of the tree constructed by EBF. The average distance from nodes to the sink is nearly the same for both algorithms. In topologies formed by 300 nodes, the difference does not exceed 7%. Figure 10 confirms that EBF ensures the quality of the constructed tree. As well as the average distance to the sink, the average number of hops to the sink is approximately the same. In none of the evaluated scenarios does the difference between the paths constructed by EBF and DBF reach one hop.

The DBF algorithm is not scalable, because there is a potential for an exponential explosion in the number of transmitted messages with the number of nodes [13]. Moreover, as the number of transmitted messages increases, there is also an increase in the proportion of such messages that do not carry route offers with an advantage larger than *α* over the previously-accepted route. Consequently, the number of messages discarded by the EBF algorithm also increases. Therefore, the increase in the number of messages for the EBF algorithm when the number of nodes increases is almost linear. However, the increase in the number of discarded messages impacts the quality of the constructed tree, and that is the reason why the differences in the path length and in the hop count also increase with the number of nodes.

## 5. Conclusions

In this paper, we proposed a novel distributed algorithm for constructing spanning trees in WSNs. The implementation and efficiency evaluation of some algorithms for constructing spanning trees found in the literature helped us to identify their advantages and drawbacks. The proposed algorithm is a more efficient and suitable solution than its counterparts in the literature, due to several factors. Firstly, EBF constructs the routing tree even in the event of message losses or node failures. This is an important feature, because message losses is a common event in wireless networks, and sensor nodes are prone to failures. Another important feature is scalability, since WSNs are frequently formed by a large number of nodes. Simulations showed that EBF significantly reduces the runtime and communication overhead of the previously proposed solutions without causing an excessive impact on the quality of the tree. In addition, EBF is flexible with respect to the metric used to compute the cost of the links, does not demand a lot of computational resources and also does not require network topology knowledge. Lastly, the constructed structure is resilient, since nodes have alternative routes to be used as mechanisms for fault tolerance and load balancing.

We identified the following issues for the continuation of this work: the proposition of a protocol for the reorganization of the routing tree, inclusion of a power consumption model, the proposition of new metrics considering the probability of an outage for computing the costs of the links and the evaluation of the network lifetime.

## Figures and Tables

**Table 1.** Main features of the algorithms: DBF, shortest hop multipath (SHM) and depth-first search (DFS).

**Features**	**DBF**	**SHM**	**DFS**
Absence of synchronization overhead	•		•
Demands few computational resources	•		
Constructs a resilient structure		•	
Lack of termination detection	•		
Inflexible with respect to the metric used to compute the cost of the links		•	•
Sensors have to know the network topology		•	•
Non-tolerant with respect to message losses or node failures		•	•
Excessive message exchange	•	•	
Non-scalable runtime		•	•

**Table 2.** Simulation parameters.

**Parameter**	**Step 1**	**Step 2**
*α*	{0.01, 0.02, 0.04, 0.06, 0.08, 0.1, 0.2, 0.4}	0.1
*N*	100	{50, 100, 150, 200, 250, 300}
*k*	{4, 6, 8, 10}	8
Transmission Range	295 m	295 m
Interference Range	887 m	887 m
